# Defective erythropoiesis caused by mutations of the thyroid hormone receptor α gene

**DOI:** 10.1371/journal.pgen.1006991

**Published:** 2017-09-14

**Authors:** Sunmi Park, Cho Rong Han, Jeong Won Park, Li Zhao, Xuguang Zhu, Mark Willingham, David M. Bodine, Sheue-yann Cheng

**Affiliations:** 1 Laboratory of Molecular Biology, the Center for Cancer Research, National Cancer Institute, Bethesda, Maryland, United States of America; 2 Hematopoiesis Section, National Human Geneome Research Institute, National Institutes of Health, Bethesda, Maryland, United States of America; Cincinnati Children's Hospital Medical Center, UNITED STATES

## Abstract

Patients with mutations of the *THRA* gene exhibit classical features of hypothyroidism, including erythroid disorders. We previously created a mutant mouse expressing a mutated TRα1 (denoted as PV; *Thra1*^PV/+^ mouse) that faithfully reproduces the classical hypothyroidism seen in patients. Using *Thra1*^PV/+^ mice, we explored how the TRα1PV mutant acted to cause abnormalities in erythropoiesis. *Thra1*^PV/+^ mice exhibited abnormal red blood cell indices similarly as reported for patients. The total bone marrow cells and erythrocytic progenitors were markedly reduced in the bone marrow of *Thra1*^PV/+^ mice. *In vitro* terminal differentiation assays showed a significant reduction of mature erythrocytes in *Thra1*^PV/+^ mice. In wild-type mice, the clonogenic potential of progenitors in the erythrocytic lineage was stimulated by thyroid hormone (T3), suggesting that T3 could directly accelerate the differentiation of progenitors to mature erythrocytes. Analysis of gene expression profiles showed that the key regulator of erythropoiesis, the *Gata-1* gene, and its regulated genes, such as the *Klf1*, *β-globin*, *dematin* genes, *CAII*, *band3 and eALAS* genes, involved in the maturation of erythrocytes, was decreased in the bone marrow cells of *Thra1*^PV/+^ mice. We further elucidated that the *Gata-1* gene was a T3-directly regulated gene and that TRα1PV could impair erythropoiesis via repression of the *Gata-1* gene and its regulated genes. These results provide new insights into how TRα1 mutants acted to cause erythroid abnormalities in patients with mutations of the *THRA* gene. Importantly, the *Thra1*^PV/+^ mouse could serve as a preclinical mouse model to identify novel molecular targets for treatment of erythroid disorders.

## Author summary

Patients with mutations of the *THRA* gene exhibit erythroid disorders. The molecular pathogenesis underlying erythroid abnormalities is poorly understood. In *Thra1*^PV/+^ mice expressing a dominant negative mutant TRα1PV, we found abnormal red blood cell indices similar to patients. Total bone marrow cells, the clonogenic potential of erythrocytic progenitors, and terminal differentiation of erythrocytes were markedly decreased in *Thra1*^PV/+^ mice. We elucidated that *Gata-1*, a key erythroid gene, was directly positively regulated by TRα1. The erythroid defects in *Thra1*^PV/+^ mice were due, at least partly, to the TRα1PV-mediated suppression of the *Gata-1* gene and its down-stream target genes. Over-expression of *Gata-1* rescued impaired terminal differentiation. Our studies elucidated molecular mechanisms by which TRα1 mutants caused erythroid disorders in patients. The present study suggests that therapies aimed at GATA1 could be tested as a potential target in treating erythroid abnormalities in patients.

## Introduction

Thyroid hormones have long been known to play an important role in erythropoiesis. Early *in vitro* studies demonstrated that L-thyroxine (T4) stimulates the biosynthesis of hemoglobin [1]. Experimental animal models also showed that T4 enhances red blood cell formation and stimulates hemoglobin synthesis [2]. Studies in humans have shown a causal association between hypothyroidism and anemia [3]. Persons with subclinical hypothyroidism had lower hemoglobin levels [4, 5] and a higher prevalence of anemia than euthyroid persons. Treatment of patients with subclinical hypothyroidism with thyroid hormone resulted in a significant increase in hemoglobin content [6] or erythropoietin levels [7]. However, how hypothyroidism results in erythroid disorders at the molecular level remains largely unknown.

Recently, patients with mutations of the thyroid hormone receptor α gene (*THRA*) have been reported to exhibit some of the classical symptoms and signs of hypothyroidism with impaired growth and delayed bone development [8–11]. These patients also display anemia. These findings suggested that erythropoietic disorders in humans are mediated by TRα1 mutants and support the critical role of TRα1 in erythropoiesis. The availability of a mouse model (*Thra1*^*PV/+*^ mice), harboring a mutated TRα1 (designated as TRα1PV) [12], has made it possible for us to elucidate the role of TRα1 mutants in erythroid disorders. TRα1PV has a C-terminal mutated sequence (398-PPFVLGSVRGLD- 409) [12], similar to the truncated C-terminal sequence in two patients (398-PPTLPRGL -405) [9]. The PV mutation was first identified from a patient with severe resistance to thyroid hormone (RTHβ), characterized by elevated thyroid hormone levels accompanied by normal TSH, short stature, goiter, and tachycardia [13]. The PV mutated sequence was targeted to the *Thra* gene at the corresponding position as in *THRB* gene to assess the functional consequence of TRα1 mutations at the time when no patients with the mutations of the *THRA* gene was discovered. *Thra1*^*PV/+*^ mice exhibit displayed phenotype of hypothyroidism as in the patients with severe growth retardation [12] and delayed bone development [12, 14, 15]. The *Thra1*^*PV/+*^ mouse has been used as a preclinical model to test the effectiveness of T4 treatment for the correction of impaired bone development due the actions of mutated TRα1 [16]. These studies further validate the usefulness of the *Thra1*^*PV/+*^ mouse to understand how mutations of the *THRA* gene result in deleterious abnormalities in patients.

In the present study, we first characterized the erythroid phenotypes in *Thra1*^*PV/+*^ mice and showed that *Thra1*^*PV/+*^ mice exhibit anemia with decreased red blood cells and reduced hemoglobin content similar to patients with mutations of the *THRA* gene [11]. We further identified the genes that were abnormally regulated by TRα1PV, resulting in defective erythropoiesis. Thus, our study has provided direct molecular evidence to show that mutations of the *THRA* gene could impair erythropoiesis and has uncovered novel molecular actions of TRα1 in the erythroid differentiation and development.

## Results

### Alterations of peripheral blood composition in *Thra1*^PV/+^ mice

**Fig 1:**
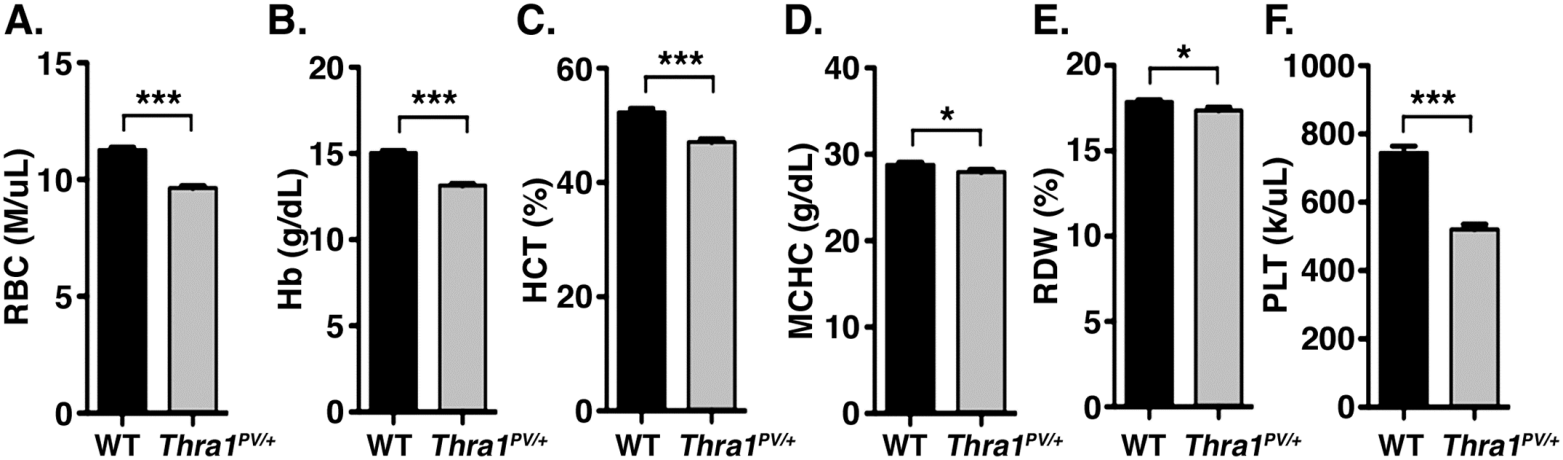
Alterations in the red blood cell indices of *Thra1*^PV/+^ mice. Comparison of complete blood counts between wild-type (WT) and *Thra1*^*PV/+*^ mice: panel A, the number of red blood cells (RBC); B, hemoglobin (Hb); C, hematocrit (HCT); D, mean corpuscular hemoglobin concentration (MCHC); E, red cell distribution width (RDW); and F, platelets (PLT) (wild type, n = 32; *Thra1*^*PV/+*^ mice, n = 31). Values are means ± SEM. Any statistical difference by *t* test versus control is indicated by p values. * denotes p<0.05; ** denotes p<0.01, *** p<0.001.

We first analyzed peripheral blood composition to characterize erythropoietic phenotypes in *Thra1*^PV/+^ mice. Complete blood count revealed that major indices for erythrocytes, such as red blood cell count (RBC), hemoglobin levels (Hb) and hematocrit (HCT), were significantly lower in *Thra1*^*PV/+*^ mice than in wild-type (WT) mice. As shown in Fig 1, RBC, Hb, and HCT were decreased by 14% (panel A), 13% (panel B), and 10% (panel C), respectively. Other erythrocyte peripheral indices, namely, the mean corpuscular hemoglobin concentration (MCHC), red cell distribution width (RDW), and platelets (PLT), were 3% (panel D), 3% (panel E), and 30% (panel F) lower in *Thra1*^PV/**+**^ mice than in WT mice. These results indicated that mutations of the *Thra* gene could affect different lineages leading to defects.

### Defective cellularity in the bone marrow of *Thra1*^PV/+^ mice

**Fig 2:**
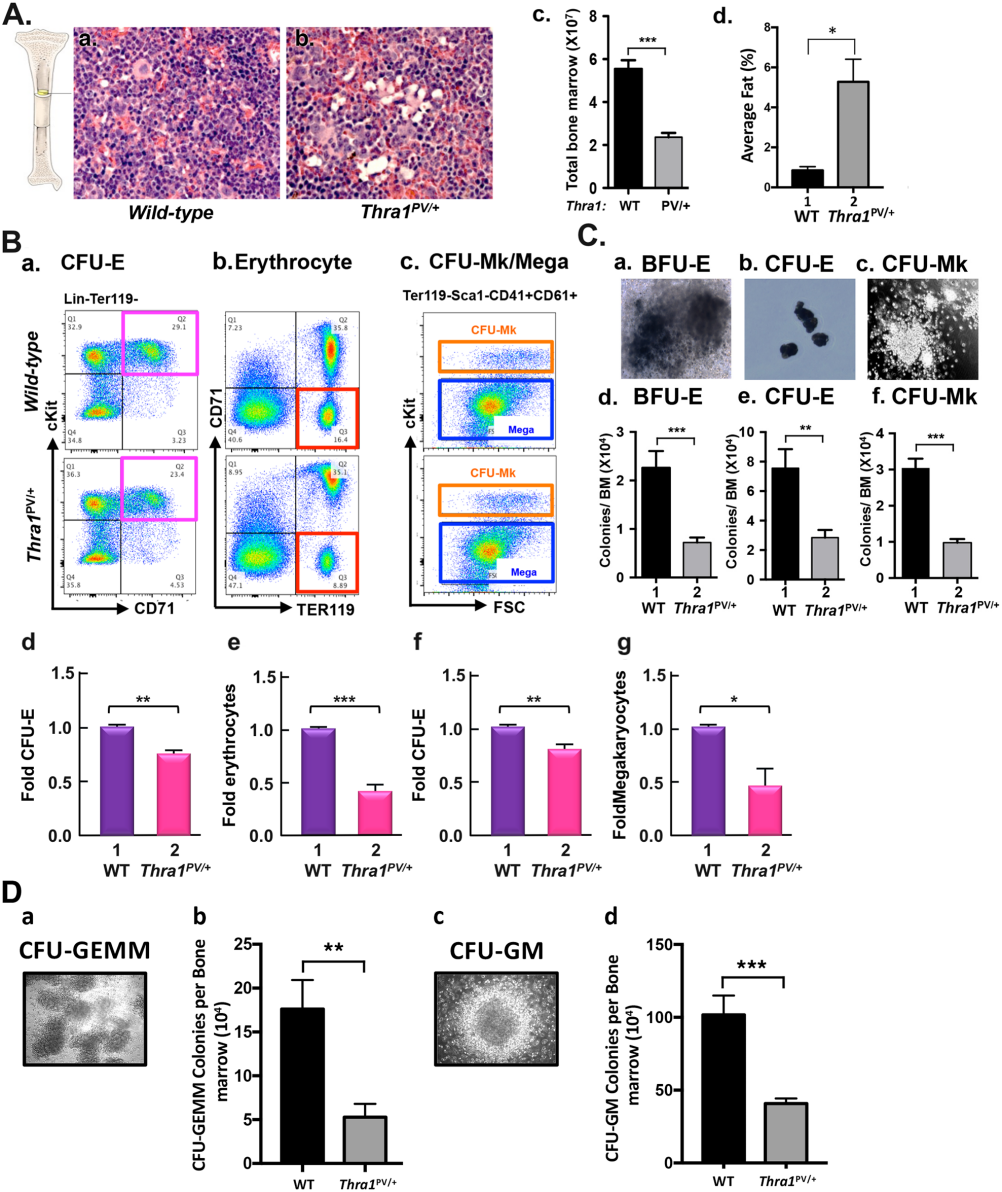
Decreased bone marrow cellularity and progenitors in *Thra1*^*PV/+*^ mice. **(A)** Hematoxylin and eosin-stained femurs sections from wild-type and *Thra1*^*PV/+*^ mice (a and b). Magnification = X330. (c). The numbers of total bone marrow cells were counted from wild-type (panel a; n = 20) and *Thra1*^PV/**+**^ mice (panel b; n = 18). (d). The fat areas in the bone marrow of wild-type mice (n = 3) and *Thra1*^*PV/+*^ mice (n = 3) shown in Figure A-a and A-b were measured by morphometry. Values are means ± SEM (n = 3). **(B)** The distribution of respective erythroid and megakaryocytic populations in the bone marrow of wild type and *Thra1*^*PV/+*^ mice. Erythroid and megakaryocytic progenitors and mature cells were analyzed by FACS using cell surface markers. CFU-E erythrocyte progenitors identified in the bone marrow cells that had characteristics of Lin-Ter119-cKit+CD71+ (the pink box in panel a). Erythrocytes identified as CD71-Ter119+ bone marrow cells (the red box in panel b). Colony-forming units-megakaryocytes (CFU-Mk) megakaryocytic progenitors identified as Ter119-Sca1-CD41+CD61+cKit+ bone marrow cells (the orange box in panel c). Megakaryocytes were identified as Ter119-Sca1-CD41+CD61+cKit- bone marrow cells (the blue box in panel c). The numbers of CFU-E cells (panel d), erythrocytes (panel e), CFU-Mk cells (panel f), and megakaryocytes (panel g) were counted and the data calculated as fold changes in *Thra1*^*PV/+*^ versus wild-type mice. The representative data from three experiments are shown. Values are means ± SEM (n = 3). The p values are shown. **(C)** *In vitro* colony forming assays of bone marrow cells from wild-type and *Thra1*^PV/**+**^ mice (panels a-c). Morphological characteristics of burst-forming units-erythroid (BFU-E) progenitors (panel a), CFU-E progenitors (panel b), and CFU-Mk progenitor (panel c) colonies by phase contrast microscopy. Panels d, e, and f show the scored colonies of the BFU-E, CFU-E, and CFU-Mk. BFU-E was counted after 14 days. CFU-E was counted after 2 days. CFU-Mk was counted after 7 days *in vitro* culturing. The data are presented as ratios of total number of colonies versus total bone marrow cells. Values are means ± SEM (duplicates in each assay; WT mice, n = 7; *Thra1*^*PV/+*^ mice, n = 6). * denotes p<0.05; ** denotes p<0.01, *** p<0.001. **(D)**. Morphological characteristics of multi-potential (CFU-GEMM) progenitor colonies (panel a), granulocyte/macrophage progenitor cells (CFU-GM) (panel c) by phase contrast microscopy (x100). Panels b and d show the number of colonies of CFU-GEMM and CFU-GM, respectively. The colonies were counted after 8 days in vitro culturing as described in Methods. The data are presented as number of colonies versus total bone marrow cells. Values are means ± SEM (WT mice, n = 4; *Thra1*^*PV/+*^ mice, n = 4; quadruplicate in each assay).

After birth and throughout adult life, the bone marrow remains the major hematopoietic organ in mice [17]. We therefore examined the histology of H & E stained femur sections to assess the cellularity of the bone marrow. As shown in Fig 2A, fewer bone marrow cells with a higher fat deposit were apparent in *Thra1*^*PV/+*^ mice than wild-type mice (Fig 2A, compare panel b with panel a). By counting cell numbers, we found that total bone marrow cells were decreased 57% in *Thra1*^*PV/+*^ mice (Fig 2A-c). We have measured the fat areas in the bone marrow of wild-type mice (n = 3) and *Thra1*^*PV/+*^ mice (n = 3). The quantitative data is now shown as Fig 2A-d, indicating that the fat (% of total area) was 6.2-fold higher in the bone marrow of *Thra1*^*PV/+*^ mice than in wild-type mice.

We next assessed which abnormal population of progenitors in the bone marrow cells of *Thra1*^*PV/+*^ mice contributed to anemia. It is known that a small population of hematopoietic stem cells (HSC) gives rise to multipotent progenitors (MPP), which subsequently differentiate into common myeloid population cells (CMP population cells) and common lymphoid progenitors (CLP). CMP is differentiated into megakaryocytic/erythroid (MEP) and granulocyte-myeloid (GMP) progenitors. Because patients with mutations of the *THRA* gene exhibit anemia, we focused on the analysis of the lineage derived from MEP, and its subsequent downstream progenitors: burst-forming unit-erythroid (BFU-E) to colony-forming unit-erythroid (CFU-E), then to erythroblasts, and ultimately to mature erythrocytes [18–20]. To assess the effect of TRα1PV mutation on the number of progenitors in the MEP lineage, we analyzed the progenitors by specific cell surface markers in the bone marrow using flow cytometry. Erythroid cells at different developmental stages can be identified by different cell surface markers: Ter119 (erythroid specific glycophorin) and CD71 (the transferrin receptor). Ter119 expression increases as maturation of erythrocyte progresses [21, 22]. CD71 is the transferrin receptor that expresses at high levels in early erythroid precursors, but its levels decrease toward erythroid maturation [21, 22]. cKit (CD117) is a cytokine receptor expressed on the surface of hematopoietic stem cells, MPP, and CMP. Stem cells antigen 1 (Sca1) is expressed in HSC [19, 23]. Using these specific cell markers, we identified which sub-populations were altered in the bone marrow cells of *Thra1*^*PV/+*^ mice.

CFU-E progenitors reside in the lineage negative (Lin-)Ter119-CD71+cKit+ populations of the bone marrow (Fig 2B, panel a). CFU-E progenitors were decreased 24% in *Thra1*^PV/+^ mice compared with wild-type mice (Fig 2B, panel d). Mature red blood cells and reticulocyte precursors exhibited characteristic Ter119+CD71- expression on the cell surface (Fig 2B, panel b). We found that erythrocytes were decreased 56% in the bone marrow of *Thra1*^*PV/+*^ mice (Fig 2B, panel e). CFU-Mk progenitors and megakaryocytes reside in Ter119-Sca1-CD41+CD61+ population (Fig 2B panel c). The only difference between CFU-Mk and megakaryocyte is that CFU-Mk expresses cKit as a marker in the progenitors. We found that CFU-Mk progenitors and megakaryocytes were decreased 19% and 52%, respectively, in the bone marrow of *Thra1*^*PV/+*^ mice (Fig 2B, panels f and g, respectively). Notably, the extent of differences between WT and *Thra1*^*PV/+*^ mice was greater in the CFU-E and erythrocytes than in CFU-Mk and megakaryocytes.

We further carried out *in vitro* colony forming unit assays. BFU-E progenitors were defined as cells that colonize after 14 days under defined-*in vitro* culture conditions (Fig 2C, panel a). CFU-E progenitors were colonized cells after 2 days in defined culture conditions (Fig 2C, panel b). CFU-Mk progenitors were counted after 7 days in defined culture conditions (Fig 2C, panel c). BFU-E, CFU-E, and CFU-Mk (expressed as relative colonies versus total bone marrow cells) were decreased 68% (Fig 2C-d), 62% (Fig 2C-e), and 68% (Fig 2C-f), respectively, in *Thra1*^*PV/+*^ mice as compared with WT mice. We further carried out colony assays for CFU-GEMM and CFU-GM. The number of CFU-GEMM and CFU-GM colonies as shown in Fig 2D-a and 2D-c, respectively, was 70% and 60% lower, respectively, in *Thra1*^*PV/+*^ mice than in WT mice (Fig 2D-b and 2D-d). These results imply that the capacity of progenitor cells to differentiate from MEP to erythroblasts as well as to megakaryocytes was impaired, leading to erythroid disorders in *Thra1*^*PV/+*^ mice.

### Stimulation of the erythroid lineage differentiation by T3

**Fig 3:**
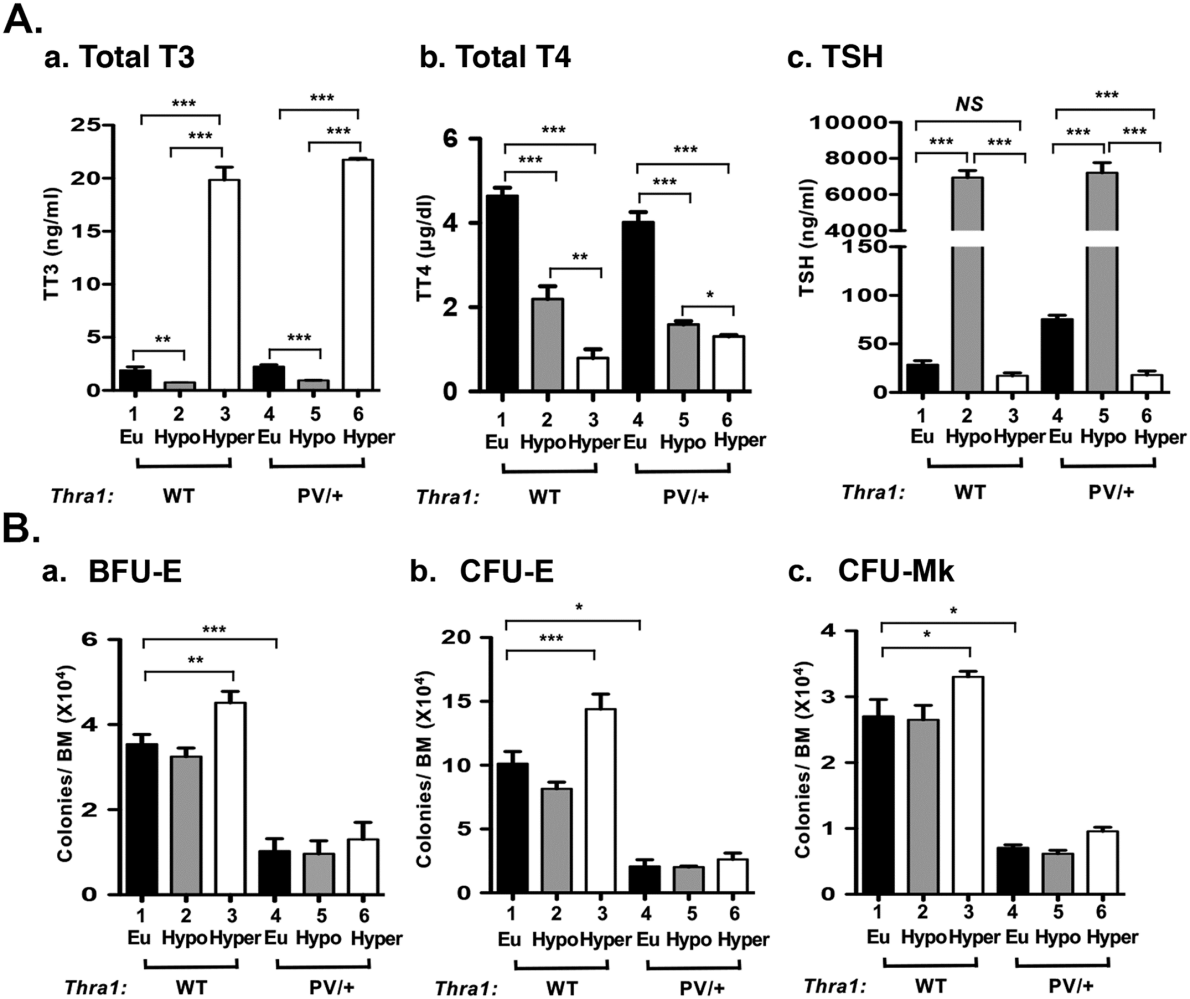
Comparison of effects of thyroid hormone on the progenitors in the megakaryocyte/erythroid lineage of wild-type and *Thra1*^*PV/+*^ mice. **(A)** Serum total T3 (panel a), total T4 (panel b), and TSH (panel c) were determined from wild type and *Thra1*^*PV/+*^ mice (each n = 3) under the euthyoid, hypothyroid and hyperthyroid condition as described in Methods. **(B)** Colonies of erythroid progenitors (BFU-E, panel a, and CFU-E, panel b) and megakaryocytic progenitors (CFU-Mk, panel c) were counted from total bone marrow of euthyroid (filled bars), hypothyroid (grey bars), and hyperthyroid (open bars) mice. Values are means ± SEM (duplicates in each assay from WT mice, n = 3; *Thra1*^*PV/+*^ mice, n = 3). * denotes p<0.05; ** denotes p<0.01; *** p<0.001.

TRα1PV is a dominantly negative mutant and cannot bind T3 [12]. That the capacity of the progenitors to form BFU-E, CFU-E, and CFU-Mk was impaired in the bone marrow of *Thra1*^*PV/+*^ mice prompted us to ascertain whether the differentiation from MEP to downstream BFU and CFU was regulated by thyroid hormones. We therefore rendered *Thra1*^*PV/+*^ mice hypothyroid by treating them with PTU and rendered the PTU-treated mice hyperthyroid by T3 treatment. As shown in Fig 3A, PTU treatment was effective in lowering serum total T3 and T4 levels (bar 2 in panels a and b, respectively) in WT mice. In line with the lowering of T3 and T4, TSH was highly elevated (bar 2 in Fig 3A-c). T3 treatment of PTU-treated WT mice led to highly elevated T3 (Fig 3A-a, bar 3) and so suppressed TSH levels (Fig 3A-c, bar 3). Treatment of *Thra1*^*PV/+*^ mice with PTU followed by T3 injection led to similar changes as shown for WT mice (Fig 3A-a, 3A-b and 3A-c, bars 4–6). These findings are consistent with the earlier reports that the feedback loop in the pituitary-thyroid axis was not affected by expressing TRα1PV mutant in *Thra1*^*PV/+*^ mice [12].

We next isolated bone marrow cells from hypothyroid (PTU-treated) and hyperthyroid (T3-treated) mice and carried out colony forming assays. No significant differences in BFU-E (Fig 3B-a, bars 1–2), CFU-E (Fig 3B-b, bars 1–2), and CFU-Mk (Fig 3B-c, bars 1–2) were detected between hypothyroid and euthyroid mice. However, T3 treatment increased the numbers of BFU-E (Fig 3B-a, bar 3), CFU-E (Fig 3B-b, bar 3), and CFU-Mk (Fig 3B-c, bar 3) by 42%, 77% and 25%, respectively from hypothyroid to hyperthyroid mice. In contrast, in *Thra1*^*PV/+*^ mice, compared to WT mice, there was markedly decreased BFU-E (Fig 3B-a), CFU-E (Fig 3B-b), and CFU-Mk (Fig 3B-c) (compare bar 4–6 to bars 1–3). Moreover, the extent of decreases in *Thra1*^*PV/+*^ mice were not affected by T3 treatment (bars 4–6). These results indicated that colony forming units derived from MEP were stimulated by T3 in WT mice, but were not affected by T3 in *Thra1*^*PV/+*^ mice due to the actions of dominant negative TRα1PV mutant.

**Fig 4:**
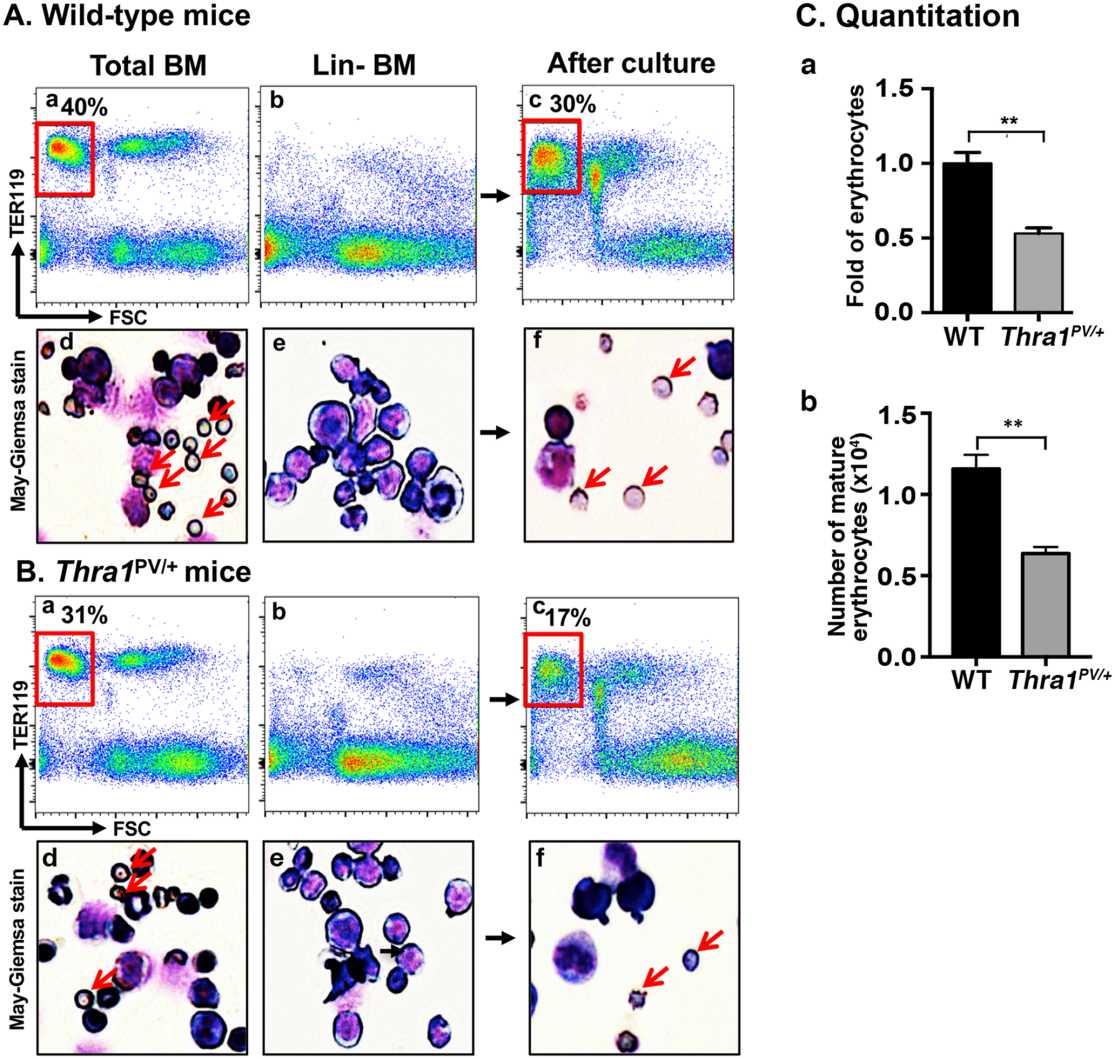
Decreased terminal erythroid differentiation in the lineage depleted bone marrow cells (Lin- BM) of *Thra1*^*PV/+*^ mice. **(A and B)** Total bone marrow profile from wild-type (A-a) and *Thra1*^*PV/+*^ mice (B-a) as determined by flow cytometry using a late erythroid surface phenotype (Ter119+FSC^low^) population is boxed in red. The corresponding May-Gr**Ü**nwald-Giemsa stained cells are shown (A-d for wild-type mice and B-d for *Thra1*^*PV/+*^ mice). Population of Lin- BM cells from wild-type (A-b) and *Thra1*^PV/+^ (B-b) mice. After 3-day culturing, Ter119+FSC^low^ population is shown boxed in red (A-c for wild-type mice and B-c for *Thra1*^*PV/+*^ mice). An arrow indicates an enucleated erythrocyte after May-Gr**Ü**nwald-Giemsa staining (A-f for wild-type mice and B-f for *Thra1*^*PV/+*^ mice). **(C-a)** Quantitative analysis shows the fold changes of erythrocytes after terminal erythroid differentiation of Lin- BM cells of wild-type and *Thra1*^PV/+^ mice. **(C-b)**. The number of matured erythrocytes from Lin-bone marrow cells of WT mice and *Thra1*^*PV/+*^ mice were also compared. Values are means ± SEM (n = 3). ** denotes p<0.01.

To further confirm that TRα1PV mutant acted to inhibit the differentiation in the erythroid lineage, we isolated lineage depleted bone marrow cells (Lin-BM) by eliminating mature lineage cells including T cells, B cells, macrophages, granulocytes, and erythrocytes. Using Lin- BM, we compared the maturation of erythrocytes in WT and *Thra1*^*PV/+*^ mice using an *in vitro* terminal erythropoiesis system [24]. Using an equal number of total bone marrow cells from WT (Fig 4A-a) and *Thra1*^*PV/+*^ mice (Fig 4B-a), we found 40% and 31%, respectively, of Ter119+ with low FSC population (boxed in red). As shown by May-Gr**Ü**nwald—Giemsa staining (Fig 4A-d and 4B-d), these populations were enriched with mature erythrocytes (marked by red arrows). After depletion of mature lineage cells as evidenced by markedly reduction of Ter119-positive cells (panel b in Fig 4A and 4B) and enucleated erythrocytes by May-Gr**Ü**nwald—Giemsa staining (panel e in Fig 4A and 4B), progenitor cells were stimulated by erythyropoietin to undergo terminal erythropoiesis [24]. After culturing for 3 days, Ter119-positive cells associated with low FSC fraction were detected (panel c in Fig 4A and 4B). The enucleated cells were visualized by May-Gr**Ü**nwald—Giemsa staining (panel f in Fig 4A and 4B). The gated Ter119-positive cells associated with low FSC fraction (boxed in red) in panel c were quantified: 30% and 17% of mature erythrocytes were detected for WT and *Thra1*^*PV/+*^ mice, respectively. These results represent a 47% reduction in the capacity of Lin- MB cells from *Thra1*^*PV/+*^ mice to mature into erythrocytes (Fig 4C).

### The expression of the *Gata1* and its regulated genes is repressed in the bone marrow of *Thra1*^*PV/+*^ mice

**Fig 5:**
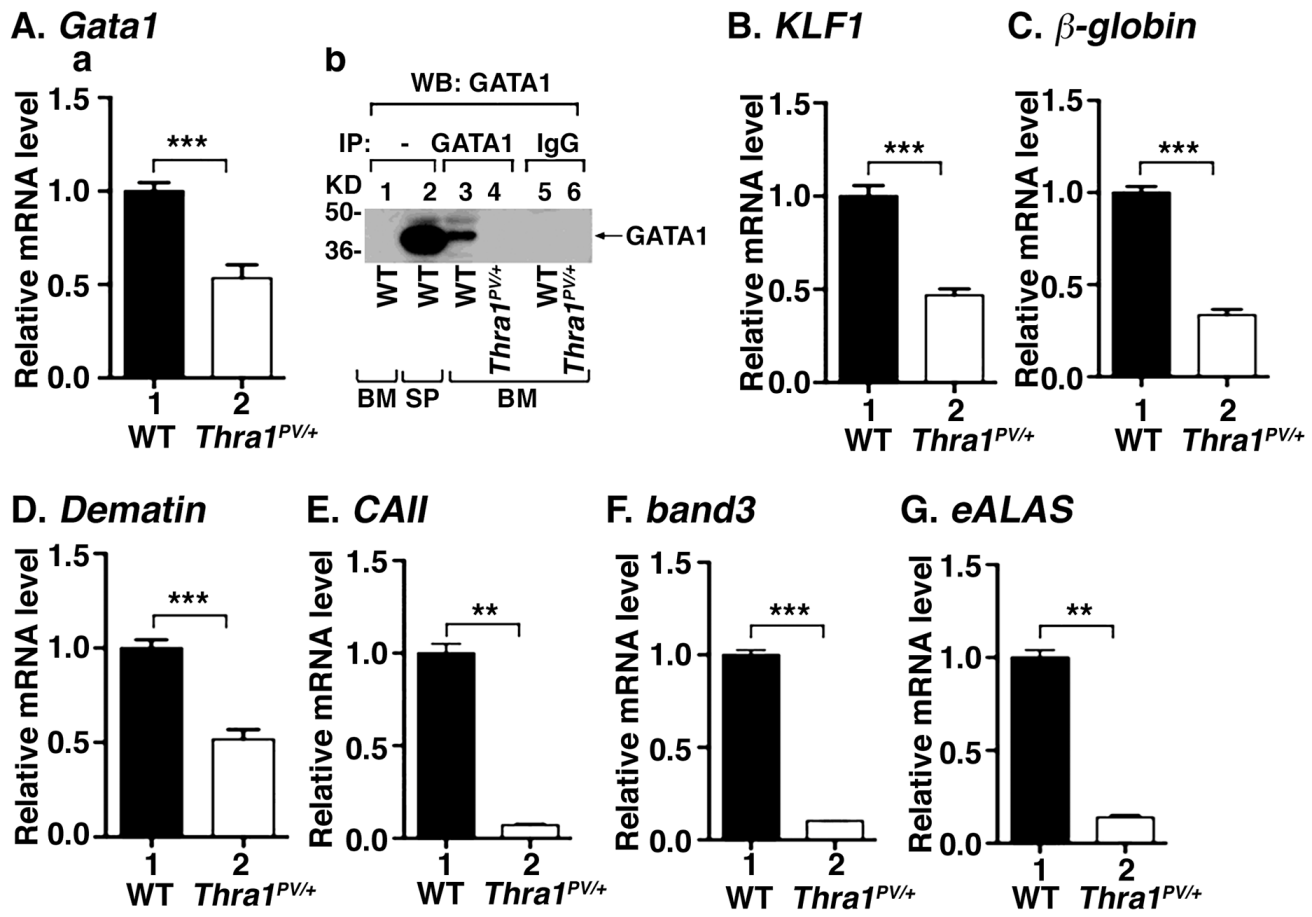
Decreased erythropoiesis related gene expression in the bone marrow of *Thra1*^PV/+^ mice. The mRNA levels of *Gata1* (panel A-a), *Klf1* (panel B), *β-globin* (panel C), *dematin* (panel D), *CA II* (panel E), *band 3* (panel F) and *eALAS* (panel G) in bone marrow cells were determined by quantitative real-time PCR. Values are means ± SEM (n = 2–4). * denotes p<0.05; ** denotes p<0.01; *** p<0.001. (A-b). Direct western blot analysis of GATA1 protein levels in the bone marrow and spleen of WT mice (lanes 1, and 2, respectively). (A-b). Analysis of the GATA1 protein levels in the bone marrow by Co-IP as described in Methods (lanes 3 and 5, WT mice; lanes 4 and 6, *Thra1*^*PV/+*^ mice). Lanes 5 and 6 were the negative controls using mouse IgG in the immunoprecipitation. BM: bone marrow; SP: spleen.

To investigate the molecular mechanisms by which TRα1PV mutant induced erythropoietic disorders, we first analyzed the expression of a major regulator of erythropoiesis, the *Gata1* gene (erythroid transcription factor; GATA-binding factor 1). GATA1 is a member of the GATA transcription factor family and is essential for erythroid development by regulating a large ensemble of genes that mediate both the development and function of red blood cells [25, 26]. The loss of *Gata1* expression leads to erythroid maturation arrest and embryonic lethality due to anemia [27–31]. We found the expression of the *Gata1* gene was ~50% lower in the bone marrow of *Thra1*^*PV/+*^ mice than in WT mice (Fig 5A-a, compare bar 2 with bar 1). Direct western blot analysis shows that the GATA1 protein level in the bone marrow was lower than that in the spleen of WT mice (compare lane 2 with lane 1, Fig 5A-b). However, when the bone marrow cell lysates of WT mice were first enriched by immunoprecipitation followed by western blot analysis (Co-IP), GATA1 proteins were detected (lane 3, Fig 5A-b). In contrast, under identical experimental conditions, GATA1 proteins were not detected (lane 4), indicating that the GATA1 protein level in the bone marrow of *Thra1*^*PV/+*^ mice was lower than that of WT mice. Lanes 5 and 6 were the corresponding negative controls in that an irrelevant IgG was used in the immunoprecipitation step. That no signals were detected in lanes 5 and 6 indicated that the protein detected by Co-IP was specific. GATA1 regulates the expression of the *Klf1* gene, which drives erythropoiesis by affecting its downstream target genes critical for maturation of erythrocytes [25, 32, 33]. Consistent with decreased expression of the *Gata1* gene, the expression of the *Klf1* gene was 53% lower in the bone marrow of *Thra1*^*PV/+*^ mice (Fig 5B, bar 2 versus bar 1). We also analyzed the expression of KLF1 and GATA1 target genes, such as the *β-globin* (beta major globin) and the *dematin* genes (erythroid membrane and cytoskeleton related gene). The β-globin protein along with α-globin makes up the most common form of hemoglobin in adult humans. Dematin is a relatively low abundance actin binding and bundling protein associated with the spectrin—actin junctions of mature erythrocytes. Dematin binds to spectrin and dynamically regulates red cell membrane mechanical function [34]. Consistent with the reduced expression of the *Klf1* gene, the expressions of the *β-globin* and *dematin* genes were also repressed by 70% and 50%, respectively, in the bone marrow of *Thra1*^*PV/+*^ mice (Fig 5C and 5D). We further analyzed the expression of *CAII*, *band 3* and *eALAS* genes, which are regulated by GATA-1 during erythropoiesis. Moreover, these three genes are known to be directly regulated by TRα1 and T3 in birds [35–37]. The expression of these three genes was inhibited 93%, 90% and 86%, respectively, in the bone marrow of *Thra1*^*PV/+*^ mice (Fig 5E, 5F and 5G, respectively). Taken together, these data indicate that suppression of *Gata-1* gene and the known T3- target genes led to impaired erythropoiesis.

### Repression of the *Gata1* gene by TRα1PV mediates erythroid disorders in *Thra1*^*PV/+*^ mice

**Fig 6:**
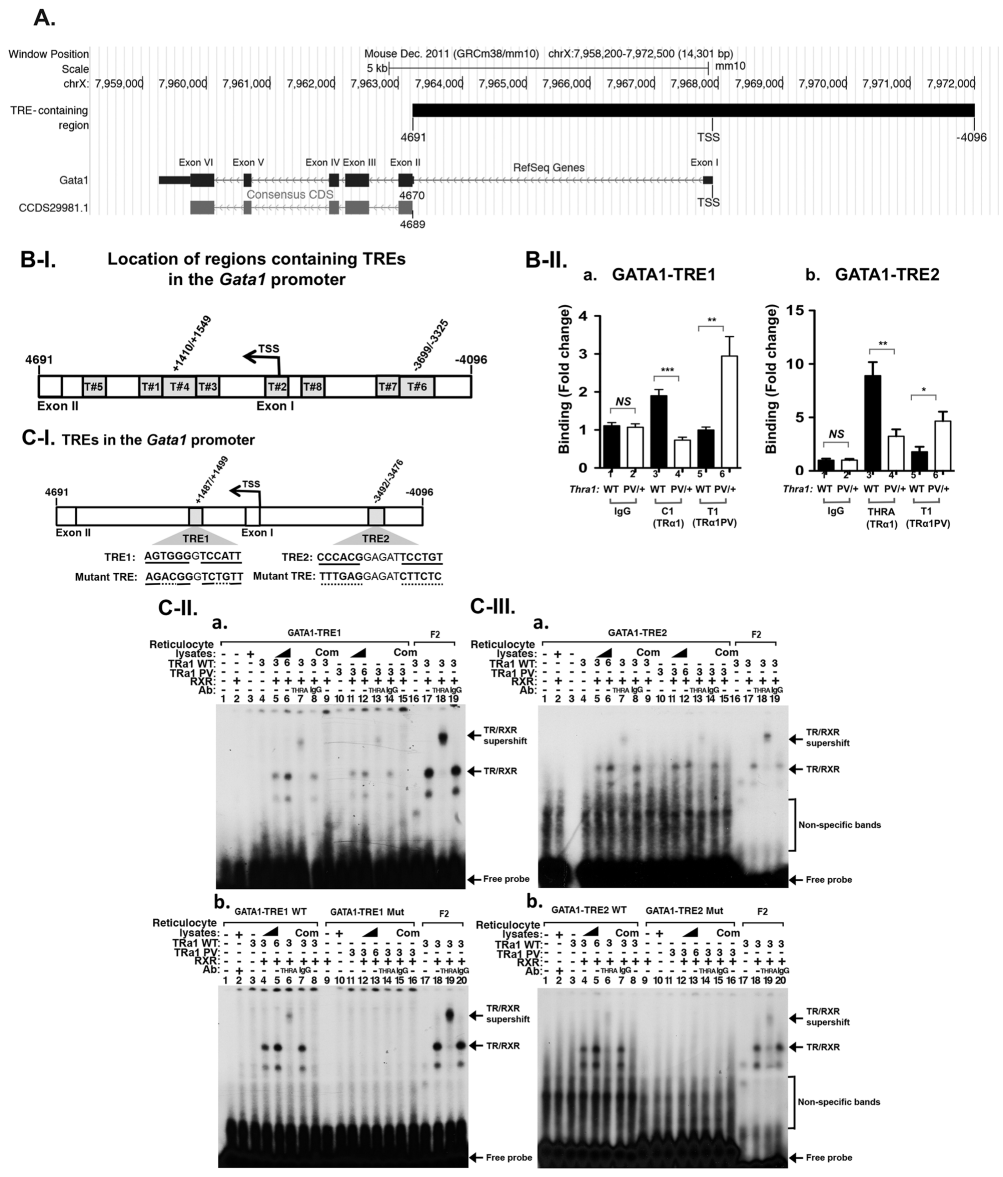
Identification of thyroid hormone response elements in the *Gata1* promoter. **(A)** Schematic representation of the mouse *Gata1* gene locus. The promoter region encompassed -4096 upstream from the transcription starting site (TSS) and the intronic sequences between exon I and exon II. The ATG was from 4689 to 4691. This figure was prepared with UCSC Genome Browser <http://genome.ucsc.edu>. **(B-I)** The putative TRE with consensus binding half sites are marked by numbers. **(B-II)** ChIP analysis in the binding of TRα1 (bars 3–4) or TRα1PV (bars 5–6) to TRE1 and TRE2 in panel a and b, respectively. IgG was used as a background control. Values are means ± SEM (n = 3). **(C-I)** The locations and sequences of wild-type and mutated TRE1 and TRE2 in the *Gata1* gene promoter. **(C-II)** EMSA analysis in the binding of TRα1 and TRα1PV to TRE1 (panel a) and to mutated TRE1 (panel b). **(C-III)** EMSA analysis in the binding of TRα1 and TRα1PV to TRE2 (panel a) and to mutated TRE2 (panel b). Lanes are marked in (C-II) and (C-III). The bands representing binding of TRE as TR/RXR heterodimers are marked by arrows.

That the expression of the *Gata1* gene was suppressed by TRα1PV prompted us to ascertain whether the *Gata1* gene was directly regulated by TR/T3. We searched for putative thyroid hormone response elements (TREs) in the GATA1 hematopoietic regulatory domain [38], located upstream of the transcription starting site (TSS) of the *Gata1* gene (Fig 6A). We also searched for putative TREs in the +4691 intronic sequence between exon I and exon II (Fig 6A). In this -4096 to +4691 region, we found putative TREs-containing regions with the core consensus sequences of the hexa-nucleotide “half-site” (A/G)GGT(C/A/G)A (T#1 –T#8, Fig 6B-I). To identify relevant TREs, we used chromatin immunoprecipitation (ChIP) to ascertain the binding of TRα1 to these eight putative TREs in the bone marrow of WT and *Thra1*^*PV/+*^ mice. When anti-TRα1 antibodies were used, a significantly higher binding of TRα1 to TRE1 (**AGTGGG**G**TCCATT** in the region +1410 to +1549 bp) were found in the bone marrow of WT mice than when anti-IgG antibodies (negative controls) were used (bar 3 versus bar 1, Fig 6B-II-a). In the bone marrow of *Thra1*^*PV/+*^ mice, when specific anti-TRα1PV antibodies (rabbit polyclonal antibody T1) were used, a significantly higher binding of TRα1PV to TRE1 than when anti-IgG antibodies (negative controls) were used (bar 6 versus bar 2, Fig 6B-II-a). Similar significantly higher binding of TRα1 (bar 3 versus bar 1, Fig 6B-II-b) and TRα1PV (bar 6 versus bar 2, Fig 6B-II-b) to TRE2 (**CCCACG**GAGAT**TCCTGT**, in the region -3699 to -3325 bp) were also detected. However, we did not find specific binding of TRα1 and TRα1PV to other regions containing putative TREs (i.e., T#1, T#2, T#3, T#5, T#7, and T#8, Fig 6B-I). These ChIP results suggested that TRα1 and TRα1PV could bind directly to these TRE-containing regions in TRE1 (region T#4) and TRE2 (region T#6) on the intronic region and the proximal promoter, respectively, of the *Gata1* gene.

We next used electrophoretic mobility shift assay (EMSA) to demonstrate directly the binding of TRα1 and TRα1PV to TREs in TRE1 and TRE2 regions (Fig 6C-I). As shown in Fig 6C-II-a, TRα1 prepared by *in vitro* transcription/translation system, bound to TRE1 (the TRE sequence is shown in Fig 6C-I) as heterodimers with the retinoid X receptor (RXR) (lanes 5 and 6 show the increasing concentrations of TRα1). The TRE1-bound TRα1 was supershifted by anti-TRα1 antibodies (lane 7), but not by control anti-IgG antibodies (lane 8). In the presence of unlabeled TRE, no binding to ^32^P-labeled TRE1 was detected (lane 9). These data indicate that TRE1 bound specifically to TRα1 as heterodimers with RXR. Similarly, TRα1PV also bound to TRE1 as heterodimers with RXR (lanes 11–12), which was supershifted by anti-TRα1PV (lane 13). No specific binding to labeled TRE in the presence of unlabeled TRE was observed (lane 15). Lanes 16–19 were positive controls using a labeled F2 (TRE with inverted two half-sites). Moreover, when TRE1 from AGTGGGGTCCATT was mutated to AGACGGGTCTGTT, no binding was detected by EMSA (Fig 6C-II-b; compare lanes 11–16 with lanes 3–8 in which wild-type TRE was used in EMSA). These EMSA results indicate that TRE1 bound directly and specifically to TRα1 and TRα1PV. Using similar EMSA, we also found specific binding of TRα1 (Fig 6C-III-a, lanes 5–9) and TRα1PV (Fig 6C-III-a, lanes 11–15) to TRE2. The binding was further confirmed by mutational analysis in which, when TRE2 was mutated from CCCACGGAGATTCCTGT to TTTGAGGAGATCTTCTC, no binding was detected by EMSA (Fig 6C-III-b; compare lanes 11–16 with lanes 3–8 in which wild-type TRE was used in EMSA). Taken together, these results indicate that we have uncovered two specific TRα1 and TRα1PV binding TREs in the promoter of the *Gata1* gene.

**Fig 7:**
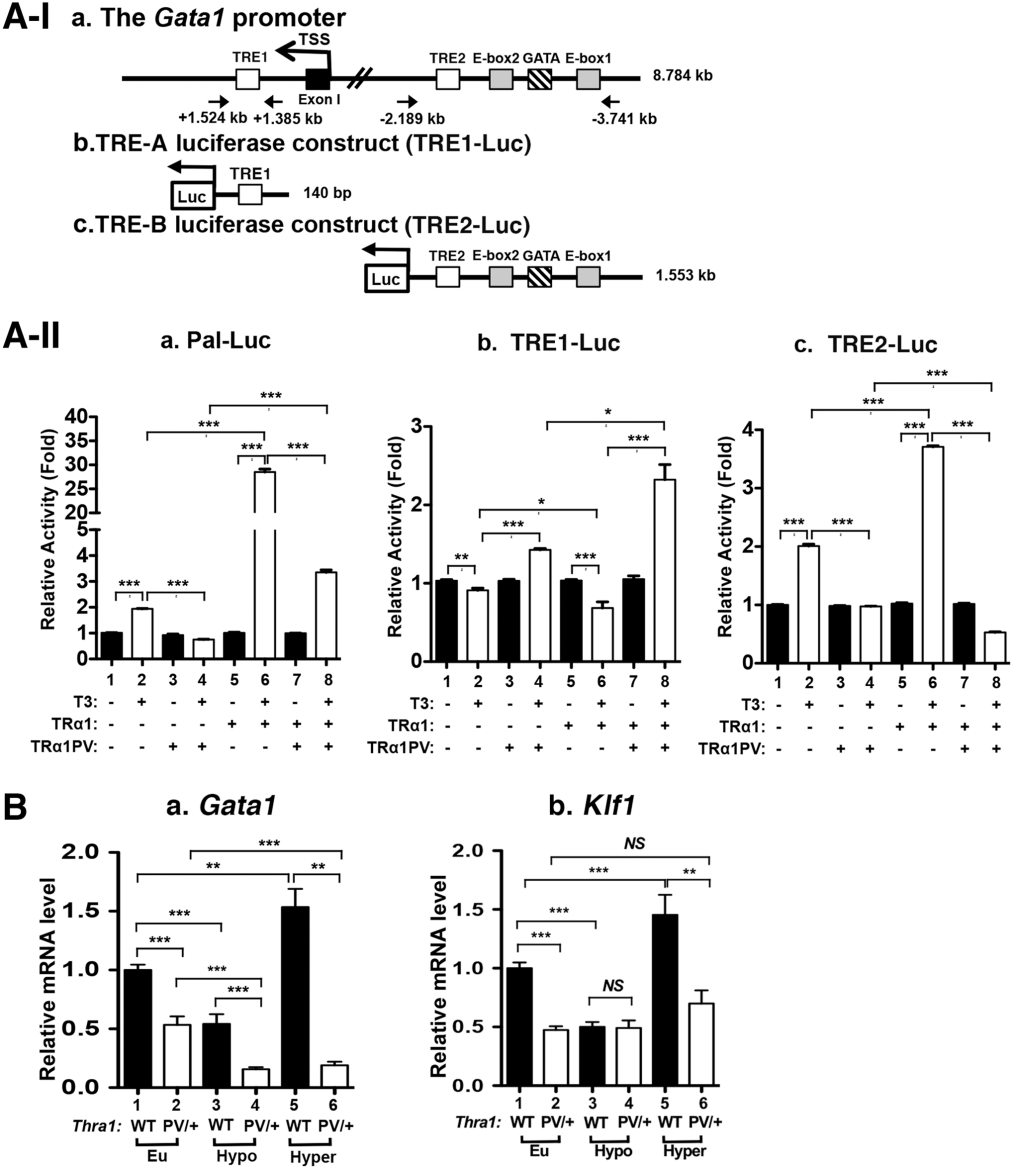
Thyroid hormone response elements in the *Gata1* promoter mediates the transcription of TRα1. Schematic representation of the *Gata1* promoter with TRE1 downstream of TSS site and TRE2 together with GATA box and two E-boxes upstream of the TSS **(A-I-a)**, the luciferase reporter construct containing TRE1 **(A-I-b)**, the luciferase reporter construct containing TRE2 **(A-I-c)**. Reporter activity mediated by a Palindromic TRE (Pal-Luc) **(A-II-a)**, TRE1 **(A-II-b)** and TRE2 **(A-II-c)** in K562 cells expressing endogenous TR (bars 1–2) and with transfected TRα1PV(bars 3–4), with cells transfected with TRα1 without TRα1PV (bars 5–6) or with TRα1PV (bars 7–8), in the absence or presence of T3. Values are means ± SEM (n = 3). **(B)** Effects of thyroid hormone on the expression of the *Gata1* (panel a, n = 4) and the *Klf1* gene (panel b; n = 3). The treatment of mice with PTU with or without T3, to make mice hypothyroid or hyperthyroid, was the same as described in Fig 3. Values are means ± SEM (n = 3). * denotes p<0.05; ** denotes p<0.01; *** p<0.001.

To assess whether these two TREs mediated the TRα1-dependent regulation of the transcription of the *Gata1* gene, we constructed reporters in which the expression of luciferase is mediated by TRE1 or TRE2. We cloned the 0.140 kb fragment containing the TRE1 (Fig 7A-I-a and 7A-I-b) and 1.553 kb fragment containing the TRE2 into the pGL4.23 luciferase plasmid (Fig 7A-I-b and 7A-I-c, respectively). It is of interest to note that in the 1.553 kb fragment, one GATA box and two E-boxes, enhancers to regulate the transcription of the *Gata1* gene, were also present [39]. These two Luc-reporters were transfected into human erythroleukemia 562 (K562) cells, which express endogenous TRα1 [40]. Indeed, using a Luc-reporter containing palindromic TRE (Pal-Luc), the reporter activity mediated by the endogenous TRα1 was detected in the presence of T3 (Fig 7A-II-a, bar 2 versus bar 1). When the TRα1PV expression vector was transfected into K562 cells, the Pal-Luc reporter activity was suppressed by dominant negative activity of TRα1PV (bar 4 versus bar 2). Transfection of the TRα1 expression plasmid led to an additional 15-fold activation of Pal-Luc reporter activity (bar 6 versus bar 2). This TRα1/T3-mediated activation was repressed by the transfection of the TRα1PV expression plasmid (bar 8 versus bar 6). These results indicate that K562 cells are a good model cell line to evaluate the activity of TRE1 and TRE2 in the regulation of the *Gata1* gene by TRα1.

In contrast to Pal-Luc, in the presence of T3, a small, but significant repression of TRE1-Luc activity was observed (Fig 7A-II-b, bar 2 versus 1). Interestingly, transfection of TRα1PV led to a 1.3-fold activation of TRE1-Luc activity (bar 4 versus 2). When TRα1 expression plasmid was transfected into K562 cells, more suppression of TRE1-Luc activity than by endogenous TRα1 was observed (bar 6 versus 2). Remarkably, co-transfection of the TRα1PV expression plasmid with TRα1 expression plasmid into K562 cells resulted in a 2.5-fold activation of TRE1-Luc activity (bar 8 versus bar 6). These results demonstrated that TRE1 mediated the negative regulation activity of TRα1. These findings are reminiscent of the regulation of the *Tshα* common subunit (*α-SU*) gene by TR/T3 in the pituitary of *Thra1*^*PV/+*^ mice [12].

In contrast to TRE1-Luc activity, the luciferase activity mediated by TRE2 was activated two-fold by T3 in K562 cells (Fig 7A-II-c, bar 2 versus bar 1). This T3-stimuated reporter activity was totally abolished by transfection of the TRα1PV expression plasmid (bar 4 versus bar 2). Transfection of the TRα1 expression plasmid led to an additional 1.9-fold activation of the reporter activity (bar 6 versus bar 2). This T3-activated reporter activity mediated by TRα1 was totally abolished by co-transfection of the TRα1PV expression plasmid (bar 8 versus bar 6). These results indicate that TRE2, via T3-TRα1, mediates the positive regulation of the *Gata1* gene transcription.

The identification of one positive TRE and one negative TRE in the promoter prompted us to assess the overall regulation by TRα1of the *Gata1* gene in the bone marrow. We rendered WT and *Thra1*^*PV/+*^ mice hypothyroid by treating them with PTU and then some PTU-treated mice with T3 to make them hyperthyroid (see also Fig 3). The expression of the *Gata1* mRNA was ~50% lower in hypothyroid WT mice than in euthyroid WT mice (Fig 7B-a, bar 3 versus bar 1). The expression of the *Gata1* mRNA was ~3-fold higher in hyperthyroid WT mice than in hypothyroid WT mice (Fig 7B-a, bar 5 versus bar 3). These results indicate that the *Gata1* gene was positively regulated by T3. In *Thra1*^*PV/+*^ mice, Fig 7B-a also shows that the expression of the *Gata1* mRNA in the bone marrow of euthyroid *Thra1*^*PV/+*^ mice was ~50% lower than that in euthyroid WT mice (bar 2 versus 1). The *Gata1* mRNA expression in hypothyroid *Thra1*^*PV/+*^ mice was also lower than that in hypothyroid WT mice (bar 4 versus 3). However, the expression of the *Gata1* mRNA in the hyperthyroid in *Thra1*^*PV/+*^ mice was not significantly increased as compared with that in hypothyroid mice (Fig 7B-a, bar 6 versus bar 4), indicating that TRα1PV expressed in the bone marrow of *Thra1*^*PV/+*^ mice has lost transcription capacity due to its inability to bind T3.

**Fig 8:**
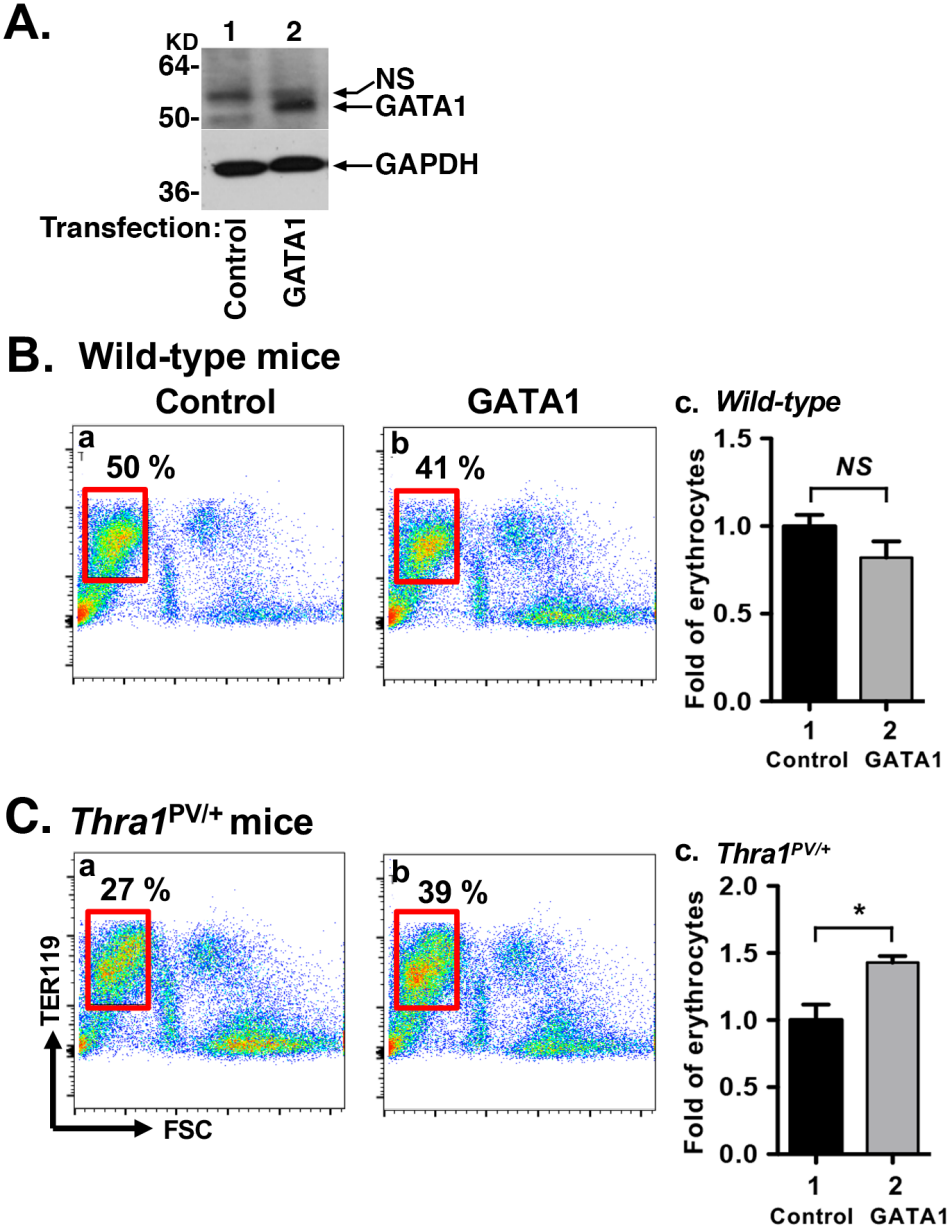
Defective terminal erythropoiesis was rescued by overexpression of the *GATA1* gene. (A) Expression of GATA1 tagged with V5 was determined in the bone marrow cells by western blot analysis using anti-V5 antibodies. Anti-GAPDH antibody served as the loading control. (B). Terminal erythropoiesis was determined by flow cytometry using a late erythroid surface phenotype (Ter119+FSC^low^, boxed in red) after transfection of control (panel a) or *GATA1* expression plasmids (panel b) in wild-type (B) and *Thra1*^*PV/+*^ mice (C). The Fold changes of matured erythrocytes after transfection of *GATA1* gene in WT mice (B, panel c) and *Thra1*^*PV/+*^ mice (C, panel c) are indicated. Values are means ± SEM (n = 3). * denotes p<0.05.

Consistent with the regulation of the *Gata1* gene by T3, similar T3-regulatory patterns in the expression of the *Klf1* gene was found in the WT mice (Fig 7B-b). The *Klf1* mRNA level was lower in the hypothyroid mice than in euthyroid mice (bar 3 versus bar 1), but was higher in hyperthyroid mice than in hypothyroid mice (Fig 7B-b, bar 5 versus bar 3). The *Klf1* mRNA level was lower in euthyroid *Thra1*^*PV/+*^ mice than in euthyroid WT mice (bar 2 versus 1). No apparent increase in the expression of the *Klf1* mRNA level was found in hyperthyroid *Thra1*^*PV/+*^ mice as compared with hypothyroid *Thra1*^*PV/+*^ mice (Fig 7B-b, bar 6 versus bar 4) due to the loss of T3 binding activity and transcriptional activity of TRα1PV. Taken together, these results indicate that the *Gata1* gene is directly positively regulated by T3/TRα1. Mutations of TRα1, such as TRα1PV, suppressed the expression of the *Gata1* gene to impair erythropoiesis in *Thra1*^*PV/+*^ mice. To further support this notion, we carried out rescue experiments. We exogenously expressed the *GATA1* gene in the Lin-bone marrow cells followed by terminal differentiation assay. As shown in Fig 8A, GATA1-tagged with V5 protein was detected by anti-V5 antibodies (lane 2), whereas it was not detected in the bone marrow cells transfected with on the control plasmid (lane 1). FACS analysis shows that exogenously expressed *GATA1* did not further increase mature erythrocytes in WT mice, as indicated by Ter119+ with low FSC population (Fig 8B-a versus 8B-b, areas boxed in red). In contrast, exogenously expressed *GATA1* led to increase in mature erythrocytes (39% in the Fig 8C-b versus 27% in the Fig 8C-a, areas boxed in red) in *Thra1*^*PV/+*^ mice. Quantitation graph represent a 43% increase in mature erythrocytes from *Thra1*^*PV/+*^ mice (Fig 8C, panel c). These data demonstrated that the defective terminal erythropoiesis from Lin-BM in *Thra1*^*PV/+*^ mice was partially corrected by exogenous expression of GATA1 protein.

**Fig 9:**
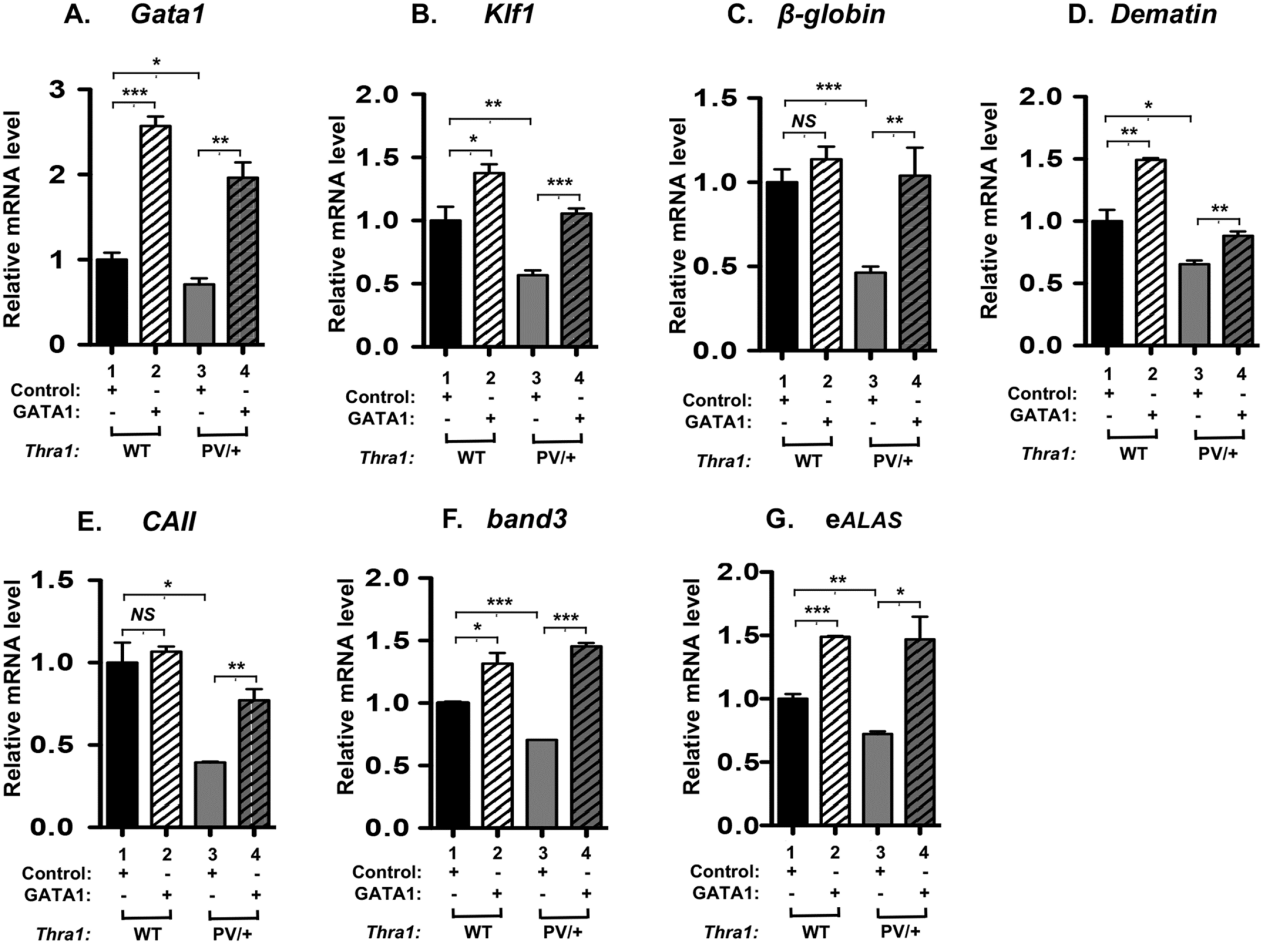
Expression of erythroid genes was increased by overexpression of the *GATA1* gene in the bone marrow of *Thra1*^*PV/+*^ mice. After transfection of V5-tagged *GATA1* plasmids into bone marrow cells, mRNA expression of erythroid genes was analyzed by quantitative real-time PCR (panel A-G). mRNA expression of *Gata1* (panel A), *Klf1* (panel B), *β-globin* (panel C), *dematin* (panel D), *CAII* (panel E), *band 3* (panel F) and *eALAS* (panel G) was increased in the bone marrow of *Thra1*^*PV/+*^ mice (lanes 4) compared with the empty vector control (lanes 3). Values are means ± SEM (n = 3). *NS*: not significant; * denotes p<0.05; ** denotes p<0.01; *** p<0.001.

We further evaluated whether the exogenously transfected *GATA1* as described above could affect the erythroid genes critical for erythropoiesis. Fig 9 shows the expression of *Gata1* (panel A), *Klf1* (panel B), *β-globin* (panel C), *dematin* (panel D), *CAII* (panel E), *band3* (panel F), and *eALAS* (panel G) were all increased by the exogenous expression of GATA1 (compare lanes 4 with lanes 3 in all panels). These results indicated that the elevated expression of these erythroid genes contributed to the partial rescue of the defective erythropoiesis and further support the essential role of GATA1 in mediating the impaired erythropoiesis in *Thra1*^*PV/+*^ mice.

## Discussion

In 2002, we created the *Thra1*^*PV/+*^ mouse that displayed aspects of the phenotype of hypothyroidism with retarded growth, delayed bone development, and marginally abnormal thyroid function tests [12]. These phenotypic manifestations are distinct from those of the *Thrb*^*PV/+*^ mice that faithfully reproduce resistance to thyroid hormone (RTH) in patients caused by mutations of the *THRB* gene [41]. The distinct phenotypic manifestations by *Thra1*^*PV/+*^ mice and *Thrb*^*PV/+*^ mice suggested TR isoform-dependent actions of TR mutants *in vivo*. However, because of the lack of patients with mutations of the *THRA* gene at the time, it was not clear whether this postulate held true. The discovery of patients with mutations of the *THRA* gene in 2012 [8], exhibiting similar hypothyroidism as in the *Thra1*^*PV/+*^ mouse, has validated that the *Thra1*^*PV/+*^ mouse is a valuable model to elucidate the molecular basis of hypothyroidism caused by mutated TRα1. Indeed, *Thra1*^*PV/+*^ mice have been used to predict the outcome of prolonged treatment with a supraphysiologic dose of T4 aimed at ameliorating the skeletal abnormalities in patients [16]. The studies provided valuable information showing that patients with different *THRA* mutations would display responses to T4 treatment that vary depending on the severity of the causative mutation.

In the present studies, we used *Thra1*^*PV/+*^ mice to understand the molecular actions of TRα1 mutants that lead to erythroid disorders in patients. We found that *Thra1*^*PV/+*^ mice exhibited abnormal red blood cell indices, decreased erythroid lineage progenitors in the bone marrow, and reduction in the terminal differentiation of progenitors in the erythroid lineage. Moreover, for the first time, we have identified a key erythropoietic gene, the *Gata1* gene, as a direct TR/T3 target gene by the discovery of thyroid hormone response elements in the promoter region of that gene. Thus, using the *Thra1*^*PV/+*^ mouse, we have provided direct evidence to indicate that mutations of TRα1 could lead to erythroid disorders and that the disorders are mediated, at least in part, by the suppressed expression of a key erythropoietic regulator, the *Gata1* gene, by TRα1 mutants. The present studies have shed new light on the molecular basis of the erythroid disorders found in patients with mutations of the *THRA* gene.

The critical role of TRα1 was also demonstrated in another mouse model, the *Thra*^*-/-*^ mouse [42]. Deficiency of TRα1 led to defective fetal and adult erythropoiesis in that erythroid progenitor numbers were decreased in fetal livers; moreover, terminal maturation of erythrocytes was impaired [42]. While the erythroid defects observed in *Thra*^*-/-*^ mice were similar to those of *Thra1*^*PV/+*^ mice, the underlying mechanisms would be different. The defective erythropoiesis in *Thra*^*-/-*^ mice would be mediated by the lack of TRα1 in regulating the transcription of erythroid-related TRα1 target genes. In contrast, in *Thra1*^*PV/+*^ mice, the impaired erythropoiesis was due to the dominant negative action of TRα1 mutants on erythroid-related TRα1 target genes, such as the suppression of the T3-positively regulated *Gata1* gene demonstrated in the present studies. At present, in *Thra*^*-/-*^ mice, the erythroid-related TRα1 target genes affected by TRα1 deficiency have not been elucidated. It is difficult to compare the extent and the scope of erythroid abnormalities from the TRα1 deficiency or mutated TRα1 until a comprehensive analysis of global gene expression profiles becomes available. Still, the similar erythroid defects observed in *Thra*^*-/-*^ mice and *Thra1*^*PV/+*^ mice clearly highlight the important role of TRα1 in erythropoiesis.

The loss of normal functions of TRα1 due to mutations as in *Thra1*^*PV/+*^ mice or due to TRα1 deficiency as in *Thra*^*-/-*^ mice could lead to erythroid disorders as shown in the present studies and in Kendrick et al. [42], respectively. However, that wild-type TRα1 plays a critical role in erythropoiesis was also demonstrated by the findings that thyroid hormones promoted the clonogenic forming ability of BFU-E, CFU-E, and CFU-Mk in hyperthyroid mice shown in the present studies (Fig 3B). These findings are consistent with the observations from clinical studies, indicating that thyroid hormones play a critical role in erythropoiesis. Qualitative and quantitative studies of erythropoiesis in patients with hyperthyroidism exhibit mild erythrocytosis with erythyroid hyperplasia and increased erythropoietic activity in the bone marrow [43]. In addition, erythrocyte counts, serum erythropoietin, and hypoxia-inducible factor 1α levels patients with untreated Graves' hyperthyroidism were significantly higher than those in the age- and sex-matched healthy controls. Methimazole or subsequent radioiodine therapy of patients with hyperthyroidism reduced erythrocytosis and thyroid function returned to normal, suggesting that thyroid hormone promotes erythrocytosis [44]. Moreover, recent population-based studies on euthyroid subjects revealed a significant positive association between thyroid hormones and erythrocyte indices, such as erythrocyte counts, and hemoglobin levels [45]; [46]. These association studies support the notion that thyroid hormones stimulate erythropoiesis. Still, the molecular mechanisms by which thyroid hormones promote erythropoiesis in hyperthyroidism are not clear. The models of *Thra1*^*PV/+*^ and *Thra*^*-/-*^ mice would provide valuable tools for such studies.

Early studies have shown that *v-erb*A is one of the two oncogenes of the avian erythroblastosis virus (AEV) [47], an acute chicken retrovirus that induces lethal erythroleukemia and sarcoma *in vivo*. V-erbA is a mutated TRα1, which acts in neoplasia by blocking erythroid differentiation and by altering the growth properties of fibroblasts [47]. Similar to TRα1PV, v-erbA functions as a transcription repressor by dominant negative interference with the transcription activity of its normal cellular homolog, c-erbA (TRα1). While the *v-erb*B locus alone is sufficient to induce erythroleukemia and sarcoma independent of the *v-erb*A gene, the *v-erb*A by itself is not capable of independently causing transformation in either erythroid cells or fibroblasts [47, 48], The expression of the *v-erb*A gene in erythroid cells blocks terminal differentiation and keeps the cells in very immature and highly proliferative stages [47]. Similar to v-erbA, TRα1PV is a potent dominant negative mutant of TRα1. These observations on the functional characteristics of v-erbA raised an important question about whether TRα1PV could act as an oncogene in *Thra1*^*PV/+*^ mice. Up to now, we have not observed any transformed phenotypes in erythroid cells of *Thra1*^*PV/+*^ mice. It is likely that another oncogene such as v-erbB (a mutated version of epidermal growth factor receptor; EGFR) would be needed, as in AEV-induced erythroleukemia and sarcoma, to collaborate with TRα1PV to bring out the transformed phenotypes in erythroid cells of *Thra1*^*PV/+*^ mice. This possibility is testable and awaits future studies.

The present studies have shown that *Thra1*^*PV/+*^ mice exhibited erythroid disorders with abnormal red blood cell indices, decreased total bone marrow cells, and reduced clonogenic potential of erythroid progenitors. The defective erythropoiesis was mediated by TRα1PV-mediated suppression of a key erythropoietic gene (the *Gata1* gene), resulting in concurrent repression of other genes involved in the maturation of erythrocytes. Two TREs were identified on the *Gata1* gene that responded to T3 differently in the reporter assay. TRα1/T3 via interacting with TRE1 mediated suppression of the transcription whereas TRα1/T3 via interacting with TRE2 activated transcription. It is of interest to point out that located upstream of TRE2 are two E-boxes and one GATA box (GATA1 gene hematopoietic enhancers) critical to the GATA1 gene transcription (see Fig 7A-I). In view of the findings that *in vivo*, T3 activated the overall transcription of the *Gata1* gene as shown in the hyperthyroid WT mice (Fig 7B-a, makes it tempting to speculate that TRE1, though shown to be negatively regulated by T3 in the reporter assay (Fig 7A-II-b), could conceivably be affected by the *GATA1* gene hematopoietic enhancers via a long-range looping mechanism, functionally acting as a positive TRE. However, interestingly, in the chicken *GATA1* promoter, a negative TRE was identified [49]. TRα1 binds to this TRE as heterodimers with the chicken ovalbumin upstream promoter transcription factor [49], suggesting that the regulatory role of TRα1 in the *GATA1* gene transcription is conserved between chicken and mouse.

The identification of the *Gata1* gene as a key regulator would suggest that the *Gata1* gene or the genes it regulates could potentially be targets for treatment. Moreover, novel TR isoform-specific thyroid hormone analogs are being developed. The *Thra1*^*PV/+*^ mouse would be a valuable model to test the effectiveness of these potential targets to correct the erythroid disorders.

## Materials and methods

### Mice and treatment

All animal studies were performed according to the approved protocols of the National Cancer Institute Animal Care and Use Committee. The animal study protocol is NCI LMB-036. Mice harboring the mutated *Thra1*^*PV*^ gene (*Thra1*^*PV*^ mice) were prepared and genotyped by PCR as described earlier [12]. Wild-type (*Thra1*^*+/+*^) and *Thra1*^*PV/+*^ female siblings were used in this study. To induce hypothyroidism, mice were fed a low-iodine diet supplemented with 0.15% propylthiouracil (LoI/PTU) (Cat# TD 95125, Harlan Teklad, Madison, WI) for 10 days. To induce hyperthyroidism, T3 (5 μg; Cat# T2752, Sigma-Aldrich, St. Louis, MO) was injected intraperitoneally to each mouse for 6 days while they were being fed with LoI/PTU diet. The same volume of vehicle (phosphate-buffered saline) was injected in the control group.

### Cells

Erythroleukemia K562 cell line was maintained in RPMI1650 (Thermo Fisher Scientific, Waltham, MA) with 10% fetal bovine serum (FBS; GE Healthcare life science, Marlborough, MA) with 50 units/ml penicillin G and 50 μg/ml streptomycin (Thermo Fisher Scientific, Waltham, MA). Bone marrow cells were isolated from femurs and tibiae of wild-type and *Thra1*^*PV*^ mice (age: 3–5 months). Single cell suspensions were prepared by passing bone marrow through a 70 μM cell strainer.

### Peripheral blood profile analysis

For analysis of complete blood counts, peripheral blood was collected in a heparinized microtube and analyzed by hematology analyzer (HEMAVET HV950FS, Drew Scientific, Miami Lakes, FL).

### Serum thyroid stimulating hormone (TSH), total T3, and T4 assays

The level of TSH in serum was measured as described [50]. Total T4 (TT4) and T3 (TT3) levels were determined by using Gamma Coat T4 and T3 assay radioimmunoassay (RIA) kits according to the manufacturer’s instruction (Cat# 06B256447 and 06B254029, MP Biomedical, LLC, Solon, OH).

### Colony assays

To detect burst-forming units-erythroid (BFU-E) colonies, 5 X 10^4^ bone marrow cells were seeded in duplicates in semisolid medium (Methocult M3434; STEMCELL Technologies, Vancouver, BC). To detect colony forming units-erythroid (CFU-E) colonies, 8 X 10^4^ bone marrow cells were seeded in duplicates in semisolid medium (Methocult M3334; STEMCELL Technologies, Vancouver, BC). To detect colony- forming units-megakaryocytes (CFU-Mk) colonies, 1 X 10^5^ bone marrow cells were seeded in duplicates in semisolid medium (Methocult-c, 04974; STEMCELL Technologies, Vancouver, BC) supplemented with 10 ng/ml Interleukin (IL)-3, 20 ng/ml Interleukin (IL)-6, 50 ng/ml thrombopoietin (TPO) (STEMCELL Technologies, Vancouver, BC). To analyze the colonies of multi-potential progenitor cells (CFU-GEMM) and granulocyte/macrophage progenitor (CFU-GM), 4 x 10^4^ bone marrow cells were mixed with semisolid medium (Methocult GF M3434; STEMCELL Technologies, Vancouver, BC) by vortexing. Bone marrow cells (4 x 10^4^ cells) from WT mice (n = 4) and *Thra1*^*PV/+*^ mice (n = 4) were seeded in 6 wells plate (DENVILLE, SCIENTIFIC INC., quadruplicates) which was cultured in 5% CO_2_ humidified incubator at 37°C. The numbers of colonies were counted under inverted microscope (Primo Vert, Ziess) by morphologic criteria 8 days after plating.

### RNA extraction and quantitative RT-PCR

Total RNA was isolated from bone marrow cells using Trizol (Thermo Fisher Scientific, Waltham, MA). RT—qPCR was performed with one step SYBR Green RT-qPCR Master Mix (Qiagen, Valencia, CA). The mRNA level of each gene was normalized to the GAPDH (glyceraldehyde-3-phosphate dehydrogenase) mRNA level. The primer sequences are listed in S1 Table.

### Chromatin immunoprecipitation assays (ChIP)

ChIP assay with bone marrow cells was performed as described previously [51]. Quantitative PCR was performed to detect the upstream fragment in *Gata1* genes (primer sequences are listed in S1 Table). The fold of changes in binding was relative to the control of IgG level as 1.

### Electrophoretic mobility-shift assay (EMSA)

Oligonucleotide probe containing mouse *Gata1* TREs or F2 TRE (positive control) was labeled with [α-32p] dCTP by Klenow fill-in reaction. Assays were performed as described previously [52].

### Plasmid constructs, and transient transfection

The *Gata1* TRE2 and *Gata1* TRE1 luciferase constructs were generated by cloning upstream *Gata1* promoter fragments into the pGL4.23 luciferase plasmid. The *Gata1-* TRE2 luciferase construct was made by insertion of a 1.553 kb XhoI-HindIII fragment representing the sequences between -3.741 kb and -2.189 kb. The *Gata1*-TRE1 luciferase construct was made by insertion of a 140 bp XhoI-HindIII fragment representing the sequences between +1.385 kb and +1.524 kb. Insert sequence validated by DNA sequencing. K562 cells were transfected with the TRE-luc reporters with Genepulse X cell electrophorators (Biorad). Bone marrow cells (2X10^6^ cells) were transfected with the GATA1-pLenti6/V5 plasmid (3 μg) provided by Dr. GP Rodgers (NHLBI; [53]) using 4D nucleofector (Lonza) in accordance with the manufacturer’s instructions. The Gata1 reporter plasmids cloned in pGL3 basic (5 μg), and the expressing plasmid for TRα1 (pcDNA3.1-TRα1; 10 μg) with or without the expression plasmid for TRα1PV (pcDNA3.1-TRα1PV; 80 μg) were transfected into K562 cells according to Guigon et al [54]. Luciferase activity was measured using Victor 3 (PerkinElmer Life and Analytical Sciences, Waltham, MA). Luciferase values were standardized to the ratio of β-galactosidase activity and protein concentration. The fold of changes in activity was based on using the values of negative control (no plasmid transfected cell without T3) as 1.

### *In vitro* terminal erythropoiesis assay

For lineage depleted bone marrow cell preparation, linage marker positive cells were depleted using the biotin based selection kit (cat# 19856, STEMCELL Technologies, Vancouver, BC) according to the manufacturer's instructions. Lin- BM cells were seeded in fibronectin-coated wells (Corning Inc, Corning, NY). To induce erythropoiesis, Lin- BM cells were cultured as described [24].

### Cytology and histochemistry

For May-Gr**Ü**nwald Giemsa stain, cytocentrifuged cells were stained with May-Gr**Ü**nwald solution (Cat# MG500, Sigma-Aldrich, St. Louis, MO) for 5 minutes and in Giemsa (Cat# GS500, Sigma-Aldrich, St. Louis, MO) for 20 minutes. For whole bone marrow sections, femurs were fixed in 10% (vol/vol) neutral buffered formalin solution (NBF, approximately 4% formaldehyde) (Sigma-Aldrich, St. Louis, MO). The embedded sections were stained with hematoxylin and eosin (HistoServ, Germantown, MD).

### Flow cytometry analysis

All antibodies used in flow cytometry were from eBiosciences (Thermo Fisher Scientific, Waltham, MA). The sources of antibodies and fluorophore-labeled antibodies used in FACS analyses are listed in S2 Table. The flow cytometry analyses were performed on a BD LSR II flow cytometer (BD bioscience, San Jose, CA) and analyzed with FloJo, LLC (Tree Star Inc, Ashland, OR).

### Western blot analysis and co-immunoprecipitation

The western blot analysis of bone marrow lysates was carried as described previously [50]. To determine the GATA1 tagged with V5 (The V5 tag is derived from a small epitope found on the P and V proteins of the paramyxovirus of simian virus 5 (SV5). after transfection with GATA1-V5 expression plasmid, anti-V5 antibodies (1:2000 dilution; Thermo Fisher Scientific) was used to detect the expressed GATA1-V5. GAPDH (1:4000 dilution; Cell Signaling Technology (Danvers, MA) was used as a loading control.

For the detection of GATA1 proteins in the bone marrow of WT and *Thra1*^*PV/+*^ mice, bone marrow lysates (600 μg each) were first immunoprecipitated with rat anti-GATA1 antibody (4 μg; Santa Crus Biotecholology, Cat.# Sc-265) or mouse IgG (4 μg; negative controls) followed by pulling down the enriched GATA1-anti-GATA1 antibody-complex with protein G-agarose beads. GATA1 proteins were subsequently detected by western blot analysis as described above using rabbit anti-GATA1 antibody (1:1000 dilution; abcam, Cat.# ab28839).

### Statistical analysis

All statistical analyses and the graphs were performed and generated using GraphPad Prism version 6.0 (GraphPad Software, La Jolla, CA). *P* < 0.05 is considered statistically significant. All data are expressed as mean ± SEM.

## Supporting information

S1 Table The primer sequences used for the determination of expression levels of the gene listed in the Table. (DOCX)

S2 Table The sources of antibodies used in FACS analysis. (DOCX)
