# Supplementary material for: Defective erythropoiesis caused by mutations of the thyroid hormone receptor α gene
Source: PLoS Genet. 2017 Sep 14;13(9):e1006991. doi: 10.1371/journal.pgen.1006991 (PMC5621702; doi:10.1371/journal.pgen.1006991)
Supplement: S1 Table — (DOCX) [file pgen.1006991.s001.docx]

**Table S1. Primer list**

**RT-qPCR Primers**

|  | **Forward primer** | **Reverse primer** |
| --- | --- | --- |
| ***Gata1*** | 5’-ATCAGCACTGGCCTACTACAGAG-3’ | 5’- GAGAGAAGAAAGGACTGGGAAAG-3’ |
| ***Klf1*** | 5’-TCTGAGGAGACGCAGGATTT-3’ | 5’-CTCGGAACCTGGAAAGTTTG-3’ |
| ***β-globi*n** | 5’-GGCAGGCTGCTGGTTGTCTA-3’ | 5’-GCCATGGGCCTTCACTTTG-3’ |
| ***Dematin*** | 5’- ACCGCATGAGGCTTGAGAGG-3’ | 5’-TCTTCTTAAGTTCGTTCCGCTTCC-3’ |
| ***CA II*** | 5’-ATACAGCAAGCACAACGGAC-3’ | 5’-GCACTGCATTGTCCTGAGAG-3’ |
| ***band 3*** | 5’-CCCGATACACCCAGGAGATC-3’ | 5’-GCGTCATGGCAAGTAGGAAG-3’ |
| ***eALAS*** | 5’-CAAGGGAGAGGAGGGTCAAG-3’ | 5’-GATGGCCTGCACATAGATGC-3’ |

**Chip-qPCR primers**

|  | **Forward primer** | **Reverse primer** |
| --- | --- | --- |
| ***Gata1*-TRE #4** | 5’ – ACTACCACATCTGCTCCCCA -3’ | 5’ – CTCTAACCAGGAGGCACAGC -3’ |
| ***Gata1*-TRE #6** | 5’- GGTCTCAAATGGAAGCCTGA -3’ | 5’ – CGGCAGAAATTGTGCATCT -3’ |
