# Supplementary material for: Defective erythropoiesis caused by mutations of the thyroid hormone receptor α gene
Source: PLoS Genet. 2017 Sep 14;13(9):e1006991. doi: 10.1371/journal.pgen.1006991 (PMC5621702; doi:10.1371/journal.pgen.1006991)
Supplement: S2 Table — (DOCX) [file pgen.1006991.s002.docx]

**Table S2. Antibody list for FACS analysis**

| **Antibody** | **Fluorochrome** | **Clone ID** | **Purpose** | **Manufacturer** | |
| --- | --- | --- | --- | --- | --- |
| Rat α-CD4 | FITC | GK1.5 | Lineage Depletion | | eBioscience |
| Rat α-IL7Ra | FITC | A7R34 | Lineage Depletion | | eBioscience |
| Rat α-CD8 | FITC | 53-6.7 | Lineage Depletion | | eBioscience |
| Rat α-Mac-1 | FITC | M1/70 | Lineage Depletion | | eBioscience |
| Rat α-Gr-1 | FITC | RB6-8C5 | Lineage Depletion | | eBioscience |
| Rat α-B220 | FITC | RA3-6B2 | Lineage Depletion | | eBioscience |
| α-cKit | APC | 2B8 | Sort | | eBioscience |
| α-CD41 | PE-Cy7 | MWReg30 | Sort | | eBioscience |
| α-CD61 | PE | 2C9.G3 | Sort | | eBioscience |
| α-Sca-1 | PerCP-Cy5.5 | D7 | Sort | | eBioscience |
| α-Ter119 | APC-780 | TER-119 | Sort | | eBioscience |
| α-CD71 | PE | R17217 | Sort | | eBioscience |
| α-CD44 | V450 | IM7 | Sort | | eBioscience |
